# Built Environment, Psychosocial Factors and Active Commuting to School in Adolescents: Clustering a Self-Organizing Map Analysis

**DOI:** 10.3390/ijerph16010083

**Published:** 2018-12-29

**Authors:** Javier Molina-García, Xavier García-Massó, Isaac Estevan, Ana Queralt

**Affiliations:** 1 Department of Teaching of Musical, Visual and Corporal Expression, University of Valencia, Avda. dels Tarongers, 4, 46022 Valencia, Spain; xavier.garcia@uv.es (X.G.-M.); isaac.estevan@uv.es (I.E.); 2 AFIPS research group, University of Valencia, 46022 Valencia, Spain; ana.queralt@uv.es; 3 HUMAG research group, University of Valencia, 46010 Valencia, Spain; 4 Department of Nursing, University of Valencia, Jaume Roig, s/n, 46010 Valencia, Spain

**Keywords:** transportation, environment design, neighborhood, walkability, physical activity, social environment, artificial neural network, clustering, cycling, health disparities

## Abstract

Although the built environment and certain psychosocial factors are related to adolescents’ active commuting to and from school (ACS), their interrelationships have not been explored in depth. This study describes these interrelationships and behavioral profiles via a self-organizing map (SOM) analysis. The sample comprised 465 adolescents from the IPEN (International Physical Activity and the Environment Network) Adolescent study in Valencia, Spain. ACS, barriers to ACS, physical self-efficacy, social support and sociodemographics were measured by questionnaire. Street-network distance to school, net residential density and street intersection density were calculated from the Geographic Information System. The clustering of the SOM outcomes resulted in eight areas or clusters. The clusters which correspond to the lowest and highest ACS levels were then explored in depth. The lowest ACS levels presented interactions between the less supportive built environments (i.e., low levels of residential density and street connectivity in the neighborhood and greater distances to school) and unfavorable psychosocial variables (i.e., low values of physical self-efficacy and medium social support for ACS) and good access to private motorized transport at home. The adolescents with the lowest ACS values exhibited high ACS environment/safety and planning/psychosocial barrier values. Future interventions should be designed to encourage ACS and change multiple levels of influence, such as individual, psychosocial and environmental factors.

## 1. Introduction

Promoting regular physical activity in the young is now a public health concern [1]. Commuting to school represents an opportunity to incorporate physical activity (i.e., walking or cycling) into adolescents’ daily routines [2]. Moreover, active commuting to and from school (ACS) has been related to several health benefits, such as healthier weight status [3], better physical fitness [4] and psychological well-being [5]. ACS also generates environmental benefits because of a significant decrease in carbon dioxide emissions or in traffic congestion, as well as social benefits due to the facilitation of social relationships in the neighborhoods [6,7].

Adolescence is a key life-stage for ACS analysis because its members become more independent in terms of mobility in the neighborhood [8]. However, different studies have indicated a decline in the use of active modes of transport to school during adolescence in many countries [9,10,11]. Research has to identify the factors with the most influence on ACS. From an ecological perspective [12,13], ACS is the consequence of multiple interaction levels between personal, psychosocial and environmental factors. The influence of environmental factors on physical activities among adolescents has attracted great interest in recent times [14,15,16], although the literature on neighborhood characteristics as a correlate of physical activity has focused mainly on analyzing adult populations [17,18,19].

Neighborhood walkability is usually evaluated by considering components such as residential density or intersection density [16,20]. Higher residential density has been previously associated with more walking for transport [21,22], which in turn provides opportunities for greater social interactions, more commercial activity and better public transportation systems. High residential density neighborhoods are believed to be related to traffic congestion that makes it more convenient to walk or take public transport, than to drive [21,22]. On the other hand, higher street connectivity is related to greater ease with which residents can actively commute to different destinations due to there being more route choices through the street-network [23]. Although some walkability components have been related to active transport in adolescents [24,25], there is an inconsistency in the associations between built-up environment and ACS among adolescents [16,26,27,28]. Considering the emerging research [27,29,30,31], psychosocial factors (e.g., physical self-efficacy perception, social support from peers or social modeling) might affect the relationship between the built environment and physical activity among adolescents. Verhoeven et al. [27] indicated that psychosocial factors seem to be more decisive than built environmental factors in choosing the mode of commuting to school. Another recent study by Wang et al. [31] showed the importance of considering interactions between psychosocial and built environmental factors in explaining adolescents’ active transportation.

According to previous research [26,32,33], the distance to school from the adolescents’ homes is one of the environmental factors that can negatively influence the relationship between built environment and ACS. Although the environment may be conducive to ACS, adolescents are less likely to actively commute to school over long distances. Socio-economic status (e.g., parental educational level) also has a relevant role in affecting the association between built environment and ACS in adolescents [16,28]. In this regard, high socio-economic status is related to better access to private vehicles (mainly cars and motorcycles) to transport youths to school [34].

There is thus a need to evaluate in depth the interrelationships between environmental and psychosocial factors and ACS among adolescents by, for example, self-organizing maps (SOM). Following a person-centered approach, this analysis establishes relationships among a diversity of input variables plotted on maps to visualize and interpret the results found in terms of the participants’ profiles. It gives the nonlinear relationships found among the input variables when there is not an expected theoretical model (i.e., built environment and psychosocial factors that promote ACS). SOM’s main advantages include: (i) an unsupervised algorithm for nonlinear models; (ii) regardless of the amount of input variables included in the analysis, it maintains its statistical power; (iii) all the neurons or nodes in the component planes are ordered into two-dimensional maps according to the distance between the neurons in their weight vectors; and (iv) visualizing the component planes facilitates the understanding of the relationships between the input variables [35]. This study thus examines the interactions of psychosocial and built environment variables with adolescents’ ACS via SOM analysis.

## 2. Materials and Methods

### 2.1. Participants and Procedure

Data was obtained from the International Physical Activity and the Environment Network (IPEN) Adolescent study, conducted in Valencia, Spain, between 2013 and 2015 [16]. A final sample of 465 adolescents (55% girls) were recruited from nine high schools to take part in this cross-sectional study [16]. Inclusion criteria were: adolescents aged 12–18 years; living in the city of Valencia; and being able to walk without assistance. Data collection occurred during the school year, and timing was balanced by season. The schools were selected to vary on objectively assessed walkability and socio-economic status (SES) of the school neighborhoods/census blocks. Each school chose the class groups that would participate in the study. The participation rate was approximately 80%. The city of Valencia is divided into 593 census blocks (considered as the smallest administrative units), which were categorized objectively into high or low walkability groups by the Geographical Information System (GIS), based on the net residential density, land use mix and intersection density (see Molina-García et al. [16] for further information). As in previous research [36], the SES was established according to the educational qualifications in every census block (data obtained from the National Statistics Institute of Spain). As a result, every census block was grouped into deciles for walkability and SES. This allowed four groups of census blocks to be established according to low (first five deciles) or high (the remaining five deciles) walkability and SES. A 2 × 2 matrix was then defined by high/low walkability and high/low SES. Prior to data collection, written parental/guardian informed consent was obtained. Approval was also obtained from the Human Research Ethics Committee at the University of Valencia (H1380699879808).

### 2.2. Measures

A paper questionnaire, completed by adolescents, was used to evaluate socio-demographic measures (i.e., gender, age, parental educational level and number of motor vehicles per licensed driver at home), active commuting behavior and psychosocial factors. The participants were asked to indicate their parents’ level of education from 1 (none) to 5 (university training). The numbers of motor vehicles and licensed drivers was also recorded for each home. Following previous research [16,37], a variable was calculated by dividing the number of vehicles by the number of licensed drivers. Built-environment variables were generated on ArcGIS 10.2 software (ESRI, Redlands, CA, USA).

#### 2.2.1. Study Outcome: Active Commuting to and from School

Modes of commuting to and from school were assessed by asking the participants: “In an average school week, on how many days do you use the following modes of transport to get to and from school?” This question was adapted from the Centers for Disease Control [38] by IPEN investigators. Response options were walk, bike, skateboard, public transit, school bus, and car. The total number of active trips per week was analyzed. This item had previously been satisfactorily used among Spanish adolescents in a previous study and had good test-retest reliability [39]. A similar questionnaire, but only for measuring the frequency of usual walking and cycling to/from school, demonstrated an acceptable reliability (percent agreement) in a study of Australian children [40]. The responses ranged from 0 to 10 trips per week.

#### 2.2.2. Built-Environment Factors

The street-network buffers within 1 km of the adolescents’ homes were defined to estimate net residential density and street intersection density. A 1-km distance is a reasonable distance for people to walk for transport [41]. Research has also shown a 1-km buffer to give more consistent associations between built environment and physical activity among adolescents [25,42]. According to Van Loon et al. [43], larger buffer sizes better explain environment–physical activity relationships. The subjects were asked to provide their home address and GIS procedures were used to calculate the street-network distance to school.

#### 2.2.3. Psychosocial Factors

Barriers to ACS were evaluated using a valid and reliable scale that includes 18 items related to environment/safety and planning/psychosocial factors [39]. This scale uses a response format of 1 (strongly disagree) to 4 (strongly agree). In the present study the internal consistency of this scale was α = 0.81.

The Physical Self-Efficacy Scale [44] was used to determine adolescents’ beliefs about perceived physical ability. Physical ability was evaluated by the 10-item subscale of the Physical Self-Efficacy Scale. This subscale showed adequate psychometric properties as well as a unidimensional structure [44]. A Likert scale from 1 (strongly disagree) to 6 (strongly agree) was used. An example item was: ‘‘I have excellent reflexes’’. In this study, the internal consistency was α = 0.84.

Social support from peers for ACS was measured by the following item: “During a typical week how often do your brothers/sisters or friends ask you to walk or bike to school or to a friend’s house?” This item was from the Spanish version [45] of the Physical Activity Family and Friends Support Scale [46]. The item was scored on a Likert scale ranging from “never” = 0 to “very often” = 4.

### 2.3. Data Analysis

The total number of active trips per week, residential density and street intersection density within a 1-km street buffer, perceived barriers to ACS, highest parental education, distance to school, perceived physical self-efficacy, number of motor vehicles per licensed driver and social support from peers for ACS were used as the input variables for the SOM analysis. The SOM was computed on the Matlab R2008a program (Mathworks Inc., Natick, Ma, USA) and the SOM toolbox (version 2.0 beta; Laboratory of computer and information science, Helsinki University of Technology, Helsinki, Finland) for Matlab [47].

The SOM analysis used to classify the participants provides profiles by their similarities in terms of the dependent variables. The process used to obtain the SOM followed a three-step procedure [48]: the first step was the construction of a neuron network. A suitable lattice size was selected for the sample size of the study (i.e., 12 × 9 neurons) to create a neural network or set of neurons represented by a value of each input variable. For that, a first approximation of the number of neurons in the lattice was calculated as the higher integer of 5 × number of cases^0.5^. Then, the ratio of the two main eigenvalues of the autocorrelation matrix of the data input was set as the ratio between the y/x map dimensions. Finally, the x and y axes dimensions were determined to approximate the final number of neurons to the number obtained with the formula and respecting the y/x-dimension ratio. The second initialization step assigned a value or weight to each neuron for each input variable by two different ways (i.e., randomized and linear initialization) [48]. The third training step modified the values or weights of the initially assigned neurons by two different training algorithms (i.e., sequential and batch) [49].

During training several factors influence the modification of the neuronal weights in each iteration. First, an input vector (i.e., a case or subject of the study) is presented to the network. Then the neurons in the lattice “compete” (i.e., compare the Euclidian distance of their weight vector and the input vector values) to win the input vectors by achieving the smallest Euclidean distance between its weight vector and the input vector. As a result, the weight vector of the winning neuron has the closest values to the cases in the neuron. All the neurons in the lattice then adapt their weight values closer to the values of the input vector [50]. The magnitude of the adaptation depends on two processes: (a) the learning ratio, which has a high value during the beginning of the training process and is gradually reduced as the training process goes on; (b) the neighbor function, which determines the adaptation of the winning neuron and the rest of the neurons. The size of the adaptation magnitude is negatively associated with the distance between the neuron and the winner. This process is repeated until the training process ends [48,50].

Taking into account that the final analysis depends on the random procedure (e.g., initialization and entry order of the input vector), the above described process was repeated 100 times to increase the odds of finding the best solution. 1600 SOM were thus obtained from the two different training methods, four neighborhood functions and two initialization methods (i.e., 100 × 2 × 4 × 2). After multiplying the quantization and topographical errors, the map with the minimum error was then chosen [48,50].

A k-means method was then used to classify the neurons into larger groups according to the input variables’ characteristics. It should be noted that in this case the data input was composed by the neuronal weights of the SOM analysis instead of the subjects’ values. The number of clusters was set to range between 2 and 10 to avoid an excessive number of profiles. The final number of clusters was the one with the lowest Davies–Bouldin index [51]. These clusters were used to describe the individuals’ characteristics according to the built environment, psychosocial factors and ACS.

The Kruskal–Wallis test was used with the neuronal weights to compare the input variables between the clusters and multiple comparisons and a Dunn–Bonferroni correction were requested if necessary. The level of significance was set to *p* = 0.05.

## 3. Results

Table 1 indicates descriptive statistics for all participants.

**Figure 1:**
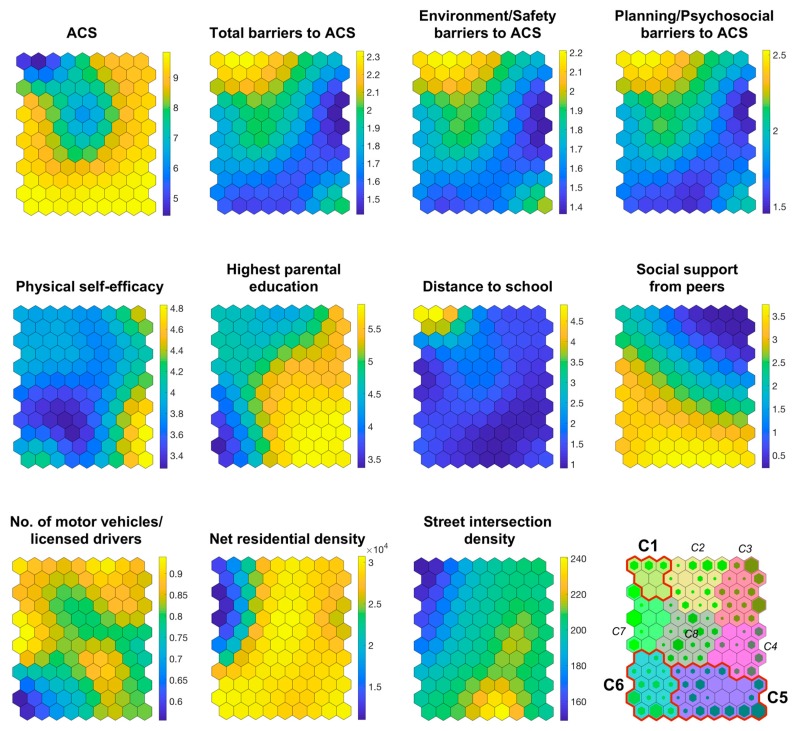
Component planes, clusters and hits obtained by the Self-Organizing Maps approach. Hits map can be seen in the right bottom corner. Empty cells show a lack of cases and greener cells indicate a large number of adolescents accumulated in them. In this map, each cluster is represented with one color and number. The eleven variables included in the analysis appear from the top to the bottom rows and from the left to the right columns. Rectangles on the right of each component map indicate the lower (blue/black) and higher (yellow/white) values of each variable. In order to understand the maps it is important to note that participants included in each neuron (hexagon) are the same in every component plane. ACS = active commuting to and from school.

Figure 1 provides the results of the SOM analysis. An explanation is provided below to facilitate its interpretation. At the end of the SOM training process, each subject (input case) is placed in a given neuron (output) according to its input variable values. This means that all the participants in a neuron share certain characteristics (i.e., input variable values). The maps shown in Figure 1 represent a network of neurons (each neuron is a hexagon) with three dimensions: the x-axis and y-axis represent the topographic situation of each neuron, so that the neurons closest together have similar weight values (i.e., input variable values) and remote neurons have very different weight vectors. 

The third axis, represented by the color map, indicates the weight value of each neuron of the network for a specific input variable. Each component plane in Figure 1 represents the value of one variable for the participants allocated to each neuron. It should be noted that the participants remain in the same neuron in all the component planes. For example, the neuron in the upper left-hand corner contains the participants with low ACS values, street intersection density and net residential density, as well as high values of total, environment/safety and planning/psychosocial barriers to ACS, number of motor vehicles per licensed drivers and distance to school.

### 3.1. Number and Description of Clusters

Figure 1 shows the clusters and hits. Each neuron cluster has a different color. The number of clusters was set at 8, based on the Davies–Bouldin index (see the component plane at the bottom right of Figure 1). Hits are described by a green hexagon in each neuron and represent the number of subjects allocated to each one. The higher the hexagon, the greater the number of participants in it.

Although the analysis reported a total of eight clusters, as in previous studies using this analysis method [52,53], we decided to describe three target clusters due to their key characteristics. Specifically, the clusters which correspond to the lowest and highest ACS levels were explored in depth. These clusters are circled in red and numbered C1, C5 and C6:

Cluster C1 is composed of the participants with low values for ACS, physical self-efficacy, net residential density and street intersection density. These adolescents also exhibited high values of total, environment/safety and planning/psychosocial barriers to ACS, distance to school and number of motor vehicles per licensed driver. The values for the highest parental education and social support from peers were medium.

Cluster C5 is formed by adolescents with high values in the following variables: ACS, parental education, social support from peers, number of motor vehicles per licensed driver, net residential density and street intersection density. A group of neurons in this cluster exhibited the highest value for physical self-efficacy. This cluster showed low values for perceived barriers to ACS and distance to school.

The participants allocated to cluster C6 presented high ACS values, net residential density and social support from peers, but low values for perceived barriers (i.e., total, environmental/safety and planning/psychosocial), physical self-efficacy, parental education, distance to school and number of motor vehicles per licensed driver. The street intersection density of this group was medium.

### 3.2. Comparison of Clusters

The Kruskal–Wallis test showed a significant cluster membership effect on all the input variables included in the SOM (Table 2). The descriptive statistics and pairwise comparisons of clusters C1, C5 and C6 are given in Table 3.

## 4. Discussion

This cross-sectional study noted that the interrelationships between the built environment and psychosocial factors explained ACS behavior in adolescents. The findings also suggest the existence of different adolescent profiles in terms of the factors that most influence them to actively travel to school. In this regard, our data concur with a recent experimental study carried out on 882 Belgian adolescents (12–16 years old) by Verhoeven et al. [54], in which the results showed that there were different subgroups of social and physical environmental preferences for active commuting by bike. In our study, the group of adolescents (i.e., cluster C1) with the lowest levels of ACS presented unfavorable levels of both environmental and psychosocial factors. In this regard, the idea that psychosocial factors seem to be more decisive in adolescents’ choice of active modes of transport [27] is not supported by the present findings. According to our data, the interactions between unfavorable psychosocial variables (e.g., low physical self-efficacy and medium social support for ACS), less supportive built environment (e.g., low levels of residential density and street connectivity and greater distances to school) and good access to private motorized transport at home explain the lowest rates of ACS. A recent study on US adolescents (aged 12–16) [31] in which an overall frequency of active transportation to neighborhood destinations (including school) was calculated, found that different two-way interactions between psychosocial factors with built environments were significant in explaining active transportation (e.g., self-efficacy x GIS-based walkability index). Our results are in line with those found for older adults [55], where the combination of psychosocial and environmental factors predicted walking behavior. Considering our evidence, multilevel interventions that improve both psychosocial factors and environmental attributes may be the most effective for adolescents.

Interestingly, the highest ACS levels in adolescents in this study can be explained through two different profiles (i.e., clusters C5 and C6). Cluster C5 justifies the high levels of ACS because of the presence of favorable psychosocial variables and supportive built environments. However, in C6, even though the subjects also showed favorable psychosocial and environmental factors, they reported low levels of physical self-efficacy. This finding is consistent with a previous study [56] on 20–64 year old adults, in which the built environmental conditions seemed to be most significant for the subjects with negative psychosocial conditions. According to the present study, improving the physical environment would be a good strategy to help adolescents with worse perceived self-efficacy in choosing active modes of transport to school. On the other hand, in our study, when the highest ACS levels are analyzed, it is notable that the peer support for ACS is always high in both C5 and C6. Significant associations between social support from peers and active transportation have been found in the literature, regardless of country and culture [31,57]. In the latter study, active transportation was positively and independently associated with social support for physical activity from peers, as well as with perceived self-efficacy. Social support for physical activity from friends was also associated with ACS in Hong Kong adolescents. According to previous research and to the present findings, social support from peers therefore seems to be a decisive factor for adolescents when choosing active modes of transport to school.

Access to private motorized transport at home showed different values in C5 and C6. As expected, the lowest access to private transport was reported in C6, whereas in C5 the values of this variable were higher. The literature indicates a significant relationship between low access to private transport, which is related to low family SES (e.g., parental education level) and active modes of commuting to school [34]. The results indicating good access to private transport in C5 are compensated by the favorable accompanying psychosocial and environmental factors. Furthermore, the short distance to school in C5 (1.1 km) may deter the use of private motorized transport. The present findings are consistent with those from a study conducted among urban Spanish adolescents, which found that the threshold distance for walking to school was 1.35 km [58].

In general, the walkability components analyzed in this study (i.e., residential density and street connectivity) have been confirmed as significant factors that support ACS in adolescents. These results contrast with previous studies that found an inconsistency in the associations between built environment and ACS in adolescents [16,26,27,28]. In a systematic review by Wong et al. [26] on the relationships between objectively measured built environment attributes and ACS in the young, only the distance to school was consistently negatively related to ACS. However, the same study did not find any consistent positive or negative relationships between ACS and residential density or intersection density. Similarly, De Meester et al. [59] found in a study of 637 Belgian 13–15 year olds that shorter distances to school, perceiving neighborhoods with connected streets or a low degree of land use mix diversity were associated with ACS and that residential density was marginally associated (*p* < 0.10) with it. The present results suggest the importance of analyzing in depth the interactions (e.g., by SOM analysis) between built environmental and psychosocial factors when studying ACS in adolescents. They also provide support for the predicted interactions between environmental and psychosocial factors indicated by ecological models [12,28].

To our knowledge, this study is one of the first to use SOM to analyze diverse objectively-measured built variables and psychosocial factors related to ACS behavior among adolescents. The strengths of the study include the use of validated self-reporting and GIS-based measures of built environments, while some of its limitations are its cross-sectional design together with the fact that we did not analyze the school built environment and that it was conducted in only one location, so that its findings may not be generalizable elsewhere. In addition, future studies should analyze (via SOM) the interactions of psychosocial and built-environment variables with walking and cycling to school as separate behaviors.

It should be noted that the study did not analyze land use mix diversity, which is a walkability component that has been positively related to ACS among adolescents [24,60]. However, the research performed to date in Europe has not found any results indicating more frequent ACS behavior in the less walkable neighborhoods (including land-use mix) [16] or with a low degree of land use mix [59]. In a recent study on Spanish adolescents [25], none of the land use analyzed (urban green-land area or commercial and entertainment areas) in the adolescents’ home environment was related to active transportation in the neighborhood. It is assumed that the proximity of different land uses encourages residents to take part in active commuting [23]. However, it does seem logical to consider that a greater diversity of destinations within proximity to adolescents’ homes would not affect ACS levels.

Findings from this study will inform policy makers and other decision makers about the significant influence of different individual, psychosocial and built environment factors on ACS. The present results can be used to design educational, encouragement and policy interventions that influence all of these factors. For instance, an effective cycling to school intervention [61] would be based, among others, on: the design of safe routes to school through non-infrastructure and infrastructure strategies [62]; a cycling skills training for adolescents [63]; and strategies that encourage not only adolescents but also their families to practice ACS [64]. In this regard, multicomponent cycling promotion programs would improve social support from peers to ACS influencing adolescents’ attitudes towards ACS, the physical self-efficacy of adolescents, the awareness of families about the positive aspects of ACS, and create supportive built environments to facilitate ACS.

## 5. Conclusions

The present findings support the theory that ACS behavior depends on the interaction of psychosocial and built environmental factors. The lowest ACS levels presented interactions between the less supportive built environment and unfavorable psychosocial variables and good access to private motorized transport at home. The adolescents with the lowest ACS values exhibited high levels of ACS barriers as regards both environment/safety and planning/psychosocial factors. According to the ecological models [12,28], future interventions to improve ACS should thus be designed to change multiple levels of influence, such as the individual, psychosocial and environmental factors and future research should focus on evaluating multilevel ACS interventions.

## Figures and Tables

**Table 1:** Descriptive statistics of the sample (n = 465).

Variable	Range	Mean (SD) or %
Gender (female)	-	55.1
Age	14–18	16.5 (0.78)
SES (highest parental education)	1–6	5.06 (1.22)
ACS (trips per week)	0–10	8.79 (3.08)

SES = socio-economic status; ACS = active commuting to school.

**Table 2:** Results of Kruskal–Wallis tests.

Variable	H (7)	*p*-Value
ACS	81.57	<0.001
Total barriers to ACS	77.15	<0.001
Environment/Safety barriers to ACS	77.23	<0.001
Planning/Psychosocial barriers to ACS	77.88	<0.001
Physical self-efficacy	34.81	<0.001
Higher parental education	88.53	<0.001
Distance to school	81.69	<0.001
Social support from peers	94.45	<0.001
No. of motor vehicles per licensed driver	59.18	<0.001
Net residential density	56.77	<0.001
Street intersection density	79.97	<0.001

ACS = active commuting to and from school.

**Table 3:** Descriptive statistics of the clusters found in this study.

Cluster	ACS (Trips per Week)	Total Barriers to ACS	Environment/Safety Barriers to ACS	Planning/Psychosocial Barriers to ACS	Physical Self-Efficacy	Higher Parental Education	Distance to School (km)	Social Support from Peers	Nº. of Motor Vehicles per Licensed Drivers	Net Residential Density (per km^2^)	Street Intersection Density (per km^2^)
1 (n = 7)	5.86	2.24	2.12	2.42	3.88	4.62	3.83	1.98	0.87	17491.18	160.62
	(1.19) ^5,6^	(0.08) ^5,6^	(0.07) ^5,6^	(0.10) ^5,6^	(0.02)	(0.04) ^5^	(0.95) ^5,6^	(0.24) ^5,6^	(0.02) ^6^	(3648.23) ^5,6^	(10.23) ^5^
2 (n = 14)	7.50	2.08	1.98	2.23	3.82	4.65	2.04	0.98	0.85	28,939.04	195.97
	(0.77)	(0.12)	(0.11)	(0.12)	(0.07)	(0.27)	(0.52)	(0.48)	(0.04)	(1545.41)	(6.82)
3 (n = 14)	8.89	1.68	1.61	1.78	4.05	5.28	1.42	0.55	0.86	27,789.62	205.77
	(0.38)	(0.16)	(0.14)	(0.20)	(0.20)	(0.18)	(0.09)	(0.35)	(0.02)	(1177.75)	(2.27)
4 (n = 12)	9.13	1.55	1.49	1.66	4.23	5.66	1.14	2.14	0.81	28,393.93	209.88
	(0.37)	(0.08)	(0.08)	(0.08)	(0.32)	(0.12)	(0.13)	(0.47)	(0.03)	(1012.26)	(3.82)
5 (n = 22)	9.50	1.67	1.65	1.70	3.95	5.68	1.11	3.40	0.81	29,140.54	215.75
	(0.35) ^1^	(0.13) ^1^	(0.12) ^1^	(0.15) ^1^	(0.53)	(0.25) ^1,6^	(0.17) ^1^	(0.26) ^1^	(0.05)	(555.91) ^1^	(11.31) ^1^
6 (n = 14)	9.43	1.67	1.62	1.75	3.70	4.03	1.39	3.39	0.70	28,833.79	200.22
	(0.36) ^1^	(0.11) ^1^	(0.10) ^1^	(0.12) ^1^	(0.29)	(0.44) ^5^	(0.12) ^1^	(0.14) ^1^	(0.09) ^1^	(2211.95) ^1^	(7.05)
7 (n = 10)	8.79	1.90	1.82	2.02	3.80	4.54	1.44	2.88	0.89	16,032.84	170.24
	(0.50)	(0.10)	(0.09)	(0.12)	(0.14)	(0.24)	(0.37)	(0.32)	(0.03)	(4152.00)	(9.74)
8 (n = 15)	7.41	1.92	1.84	2.04	3.60	5.27	1.71	2.43	0.82	29,194.15	199.54
	(0.64)	(0.10)	(0.08)	(0.12)	(0.16)	(0.29)	(0.16)	(0.46)	(0.02)	(1639.97)	(5.92)
Total (n = 108)	8.52	1.80	1.74	1.90	3.88	5.05	1.60	2.30	0.82	26,855.30	199.19
	(1.20)	(0.23)	(0.21)	(0.27)	(0.35)	(0.63)	(0.74)	(1.09)	(0.07)	(4873.48)	(17.59)

Data are expressed as mean (standard error of the mean). ACS = active commuting to and from school. n = number of neurons in the cluster. Superscript numbers indicate significant differences with this cluster (*p* < 0.05); the pairwise comparisons of clusters C1^(1)^, C5^(5)^ and C6^(6)^ are given in the table.

## References

[1] World Health Organization (2018). Global Action Plan on Physical Activity 2018–2030: More Active People for a Healthier World.

[2] Chillón P., Ortega F.B., Ruiz J.R., Veidebaum T., Oja L., Mäestu J., Sjöström M. (2010). Active commuting to school in children and adolescents: An opportunity to increase physical activity and fitness. Scand. J. Public Health.

[3] Lubans D.R., Boreham C.A., Kelly P., Foster C.E. (2011). The relationship between active travel to school and health-related fitness in children and adolescents: A systematic review. Int. J. Behav. Nutr. Phys. Act..

[4] Villa-González E., Ruiz J.R., Chillón P. (2015). Associations between active commuting to school and health-related physical fitness in Spanish school-aged children: A cross-sectional study. Int. J. Environ. Res. Public Health.

[5] Ruiz-Ariza A., de la Torre-Cruz M.J., Redecillas-Peiró M.T., Martínez-López E.J. (2015). Influence of active commuting on happiness, well-being, psychological distress and body shape in adolescents. Gac. Sanit..

[6] Frank L.D., Greenwald M.J., Winkelman S., Chapman J., Kavage S. (2010). Carbonless footprints: Promoting health and climate stabilization through active transportation. Prev. Med..

[7] Giles-Corti B., Foster S., Shilton T., Falconer R. (2010). The co-benefits for health of investing in active transportation. N. S. W. Public Health Bull..

[8] Giles-Corti B., Kelty S., Zubrick S., Villanueva K. (2009). Encouraging walking for transport and physical activity in children and adolescents: How important is the built environment?. Sports Med..

[9] Buliung R.N., Mitra R., Faulkner G. (2009). Active school transportation in the Greater Toronto Area, Canada: An exploration of trends in space and time (1986–2006). Prev. Med..

[10] Chillón P., Martínez-Gómez D., Ortega F.B., Pérez-López I.J., Díaz L.E., Veses A.M., Veiga O.L., Marcos A., Delgado-Fernández M. (2013). Six-year trend in active commuting to school in Spanish adolescents. The AVENA and AFINOS Studies. Int. J. Behav. Med..

[11] Dygrýn J., Mitáš J., Gába A., Rubín L., Frömel K. (2015). Changes in Active Commuting to School in Czech Adolescents in Different Types of Built Environment across a 10-Year Period. Int. J. Environ. Res. Public Health.

[12] Sallis J.F., Cervero R.B., Ascher W., Henderson K.A., Kraft M.K., Kerr J. (2006). An Ecological Approach to Creating Active Living Communities. Annu. Rev. Public Health.

[13] Sallis J.F., Owen N., Glanz K., Rimer B.K., Viswanath K. (2015). Ecological models of health behavior. Health Behavior: Theory, Research, and Practice.

[14] McGrath L.J., Hopkins W.G., Hinckson E.A. (2015). Associations of objectively measured built-environment attributes with youth moderate-vigorous physical activity: A systematic review and meta-analysis. Sports Med. Auckl. NZ.

[15] Lord S., Manlhiot C., Tyrrell P.N., Dobbin S., Gibson D., Chahal N., Stearne K., Fisher A., McCrindle B.W. (2015). Lower socioeconomic status, adiposity and negative health behaviours in youth: A cross-sectional observational study. BMJ Open.

[16] Molina-García J., Queralt A., Adams M.A., Conway T.L., Sallis J.F. (2017). Neighborhood built environment and socio-economic status in relation to multiple health outcomes in adolescents. Prev. Med..

[17] Adams M.A., Sallis J.F., Kerr J., Conway T.L., Saelens B.E., Frank L.D., Norman G.J., Cain K.L. (2011). Neighborhood environment profiles related to physical activity and weight status: A latent profile analysis. Prev. Med..

[18] Christiansen L.B., Madsen T., Schipperijn J., Ersbøll A.K., Troelsen J. (2014). Variations in active transport behavior among different neighborhoods and across adult lifestages. J. Transp. Health.

[19] Sallis J.F., Cerin E., Conway T.L., Adams M.A., Frank L.D., Pratt M., Salvo D., Schipperijn J., Smith G., Cain K.L. (2016). Physical activity in relation to urban environments in 14 cities worldwide: A cross-sectional study. Lancet.

[20] Frank L.D., Sallis J.F., Saelens B.E., Leary L., Cain K., Conway T.L., Hess P.M. (2010). The development of a walkability index: Application to the Neighborhood Quality of Life Study. Br. J. Sports Med..

[21] Forsyth A., Oakes J.M., Schmitz K.H., Hearst M. (2007). Does Residential Density Increase Walking and Other Physical Activity?. Urban Stud..

[22] Chaudhury H., Mahmood A., Michael Y.L., Campo M., Hay K. (2012). The influence of neighborhood residential density, physical and social environments on older adults’ physical activity: An exploratory study in two metropolitan areas. J. Aging Stud..

[23] Handy S.L., Boarnet M.G., Ewing R., Killingsworth R.E. (2002). How the built environment affects physical activity: Views from urban planning. Am. J. Prev. Med..

[24] Carlson J.A., Saelens B.E., Kerr J., Schipperijn J., Conway T.L., Frank L.D., Chapman J.E., Glanz K., Cain K.L., Sallis J.F. (2015). Association between neighborhood walkability and GPS-measured walking, bicycling and vehicle time in adolescents. Health Place.

[25] Queralt A., Molina-García J. Physical activity and active commuting in relation to objectively measured built environment attributes among adolescents. J. Phys. Act. Health.

[26] Wong B.Y.-M., Faulkner G., Buliung R. (2011). GIS measured environmental correlates of active school transport: A systematic review of 14 studies. Int. J. Behav. Nutr. Phys. Act..

[27] Verhoeven H., Simons D., Van Dyck D., Van Cauwenberg J., Clarys P., De Bourdeaudhuij I., de Geus B., Vandelanotte C., Deforche B. (2016). Psychosocial and Environmental Correlates of Walking, Cycling, Public Transport and Passive Transport to Various Destinations in Flemish Older Adolescents. PLoS ONE.

[28] Sallis J.F., Conway T.L., Cain K.L., Carlson J.A., Frank L.D., Kerr J., Glanz K., Chapman J.E., Saelens B.E. (2018). Neighborhood built environment and socioeconomic status in relation to physical activity, sedentary behavior, and weight status of adolescents. Prev. Med..

[29] Aznar S., Queralt A., García-Massó X., Villarrasa-Sapiña I., Molina-García J. (2018). Multifactorial combinations predicting active vs inactive stages of change for physical activity in adolescents considering built environment and psychosocial factors: A classification tree approach. Health Place.

[30] De Meester F., Van Dyck D., De Bourdeaudhuij I., Deforche B., Cardon G. (2013). Do psychosocial factors moderate the association between neighborhood walkability and adolescents’ physical activity?. Soc. Sci. Med..

[31] Wang X., Conway T.L., Cain K.L., Frank L.D., Saelens B.E., Geremia C., Kerr J., Glanz K., Carlson J.A., Sallis J.F. (2017). Interactions of psychosocial factors with built environments in explaining adolescents’ active transportation. Prev. Med..

[32] Panter J.R., Jones A.P., van Sluijs E.M. (2008). Environmental determinants of active travel in youth: A review and framework for future research. Int. J. Behav. Nutr. Phys. Act..

[33] Chillón P., Panter J., Corder K., Jones A.P., Van Sluijs E.M.F. (2015). A longitudinal study of the distance that young people walk to school. Health Place.

[34] Molina-García J., Queralt A. (2017). Neighborhood Built Environment and Socio-Economic Status in Relation to Active Commuting to School in Children. J. Phys. Act. Health.

[35] Herrero-Herrero M., García-Massó X., Martínez-Corralo C., Prades-Piñón J., Sanchis-Alfonso V. (2017). Relationship between the practice of physical activity and quality of movement in adolescents: A screening tool using self-organizing maps. Phys. Sportsmed..

[36] Janssen E., Sugiyama T., Winkler E., de Vries H., te Poel F., Owen N. (2010). Psychosocial correlates of leisure-time walking among Australian adults of lower and higher socio-economic status. Health Educ. Res..

[37] Estevan I., Queralt A., Molina-García J. (2018). Biking to School: The Role of Bicycle-Sharing Programs in Adolescents. J. Sch. Health.

[38] Centers for Disease Control (CDC) Kids-Walk-to-School Program. http://www.cdc.gov/nccdphp/dnpa/kidswalk/resources.htm.

[39] Molina-García J., Queralt A., Estevan I., Álvarez O., Castillo I. (2016). Perceived barriers to active commuting to school: Reliability and validity of a scale. Gac. Sanit..

[40] Timperio A., Ball K., Salmon J., Roberts R., Giles-Corti B., Simmons D., Baur L.A., Crawford D. (2006). Personal, family, social, and environmental correlates of active commuting to school. Am. J. Prev. Med..

[41] Villanueva K., Knuiman M., Nathan A., Giles-Corti B., Christian H., Foster S., Bull F. (2014). The impact of neighborhood walkability on walking: Does it differ across adult life stage and does neighborhood buffer size matter?. Health Place.

[42] Bentley R., Blakely T., Kavanagh A., Aitken Z., King T., McElwee P., Giles-Corti B., Turrell G. (2018). A Longitudinal Study Examining Changes in Street Connectivity, Land Use, and Density of Dwellings and Walking for Transport in Brisbane, Australia. Environ. Health Perspect..

[43] Van Loon J., Frank L.D., Nettlefold L., Naylor P.-J. (2014). Youth physical activity and the neighbourhood environment: Examining correlates and the role of neighbourhood definition. Soc. Sci. Med..

[44] Ryckman R.M., Robbins M.A., Thornton B., Cantrell P. (1982). Development and validation of a physical self-efficacy scale. J. Pers. Soc. Psychol..

[45] Molina-García J., Queralt A., Castillo I., Sallis J.F. (2015). Changes in Physical Activity Domains during the Transition out of High School: Psychosocial and Environmental Correlates. J. Phys. Act. Health.

[46] Norman G.J., Sallis J.F., Gaskins R. (2005). Comparability and reliability of paper- and computer-based measures of psychosocial constructs for adolescent physical activity and sedentary behaviors. Res. Q. Exerc. Sport.

[47] Vesanto J., Himberg J., Alhoniemi E., Parhankangas J. Self-organizing map in Matlab: The SOM Toolbox. Proceedings of the Matlab DSP Conference.

[48] Pellicer-Chenoll M., Garcia-Massó X., Morales J., Serra-Añó P., Solana-Tramunt M., González L.-M., Toca-Herrera J.-L. (2015). Physical activity, physical fitness and academic achievement in adolescents: A self-organizing maps approach. Health Educ. Res..

[49] Oliver E., Vallés Pérez I., Rivera B., María R., Martí C.I., Josep A., Botella Arbona C., Soria Olivas E. (2016). Visual data mining with self-organizing maps for “self-monitoring” data analysis. Sociol. Methods Res..

[50] Estevan I., García-Massó X., Molina-García J., Barnett L.M. (2019). Identifying profiles of children at risk of being less physically active: An exploratory study using a self-organised map approach for motor competence. J. Sports Sci..

[51] Davies D.L., Bouldin D.W. (1979). A cluster separation measure. IEEE Trans. Pattern Anal. Mach. Intell..

[52] Valencia-Peris A., Devís-Devís J., García-Massó X., Lizandra J., Pérez-Gimeno E., Peiró-Velert C. (2016). Competing Effects Between Screen Media Time and Physical Activity in Adolescent Girls: Clustering a Self-Organizing Maps Analysis. J. Phys. Act. Health.

[53] Peiró-Velert C., Valencia-Peris A., González L.M., García-Massó X., Serra-Añó P., Devís-Devís J. (2014). Screen media usage, sleep time and academic performance in adolescents: Clustering a self-organizing maps analysis. PLoS ONE.

[54] Verhoeven H., Ghekiere A., Van Cauwenberg J., Van Dyck D., De Bourdeaudhuij I., Clarys P., Deforche B. (2018). Subgroups of adolescents differing in physical and social environmental preferences towards cycling for transport: A latent class analysis. Prev. Med..

[55] Carlson J.A., Sallis J.F., Conway T.L., Saelens B.E., Frank L.D., Kerr J., Cain K.L., King A.C. (2012). Interactions between psychosocial and built environment factors in explaining older adults’ physical activity. Prev. Med..

[56] Ding D., Sallis J.F., Conway T.L., Saelens B.E., Frank L.D., Cain K.L., Slymen D.J. (2012). Interactive effects of built environment and psychosocial attributes on physical activity: A test of ecological models. Ann. Behav. Med. Publ. Soc. Behav. Med..

[57] Barnett A., Sit C.H.P., Mellecker R.R., Cerin E. (2018). Associations of socio-demographic, perceived environmental, social and psychological factors with active travel in Hong Kong adolescents: The iHealt(H) cross-sectional study. J. Transp. Health.

[58] Rodríguez-López C., Salas-Fariña Z.M., Villa-González E., Borges-Cosic M., Herrador-Colmenero M., Medina-Casaubón J., Ortega F.B., Chillón P. (2017). The Threshold Distance Associated with Walking from Home to School. Health Educ. Behav..

[59] De Meester F., Van Dyck D., De Bourdeaudhuij I., Deforche B., Cardon G. (2013). Does the perception of neighborhood built environmental attributes influence active transport in adolescents?. Int. J. Behav. Nutr. Phys. Act..

[60] Ding D., Sallis J.F., Kerr J., Lee S., Rosenberg D.E. (2011). Neighborhood environment and physical activity among youth a review. Am. J. Prev. Med..

[61] Larouche R., Mammen G., Rowe D.A., Faulkner G. (2018). Effectiveness of active school transport interventions: A systematic review and update. BMC Public Health.

[62] Henderson S., Tanner R., Klanderman N., Mattera A., Martin Webb L., Steward J. (2013). Safe routes to school: A public health practice success story—Atlanta, 2008–2010. J. Phys. Act. Health.

[63] Mandic S., Flaherty C., Ergler C., Kek C.C., Pocock T., Lawrie D., Chillón P., García Bengoechea E. (2018). Effects of cycle skills training on cycling-related knowledge, confidence and behaviour in adolescent girls. J. Transp. Health.

[64] Buckley A., Lowry M.B., Brown H., Barton B. (2013). Evaluating safe routes to school events that designate days for walking and bicycling. Transp. Policy.

